# Low pre-transplant levels of mannose-binding lectin are associated with viral infections and mortality after haematopoietic allogeneic stem cell transplantation

**DOI:** 10.1186/s12865-019-0318-8

**Published:** 2019-11-09

**Authors:** M. Puente, C. Fariñas-Alvarez, A. Moreto, P. Sánchez-Velasco, J. G. Ocejo-Vinyals, M. C. Fariñas, on behalf of SCT team

**Affiliations:** 1 Service of Hematology, Hospital Universitario Marqués de Valdecilla, Santander, Spain; 2 Present address: Service of Hematology, Hospital de Cruces, Bilbao, Spain; 3 Division of Health Care Quality, Hospital Universitario Marqués de Valdecilla, Santander, Spain; 4 Infectious Diseases Unit, Hospital Universitario Marqués de Valdecilla, IDIVAL, University of Cantabria, Av. Valdecilla s/n, 39008 Santander, Spain

**Keywords:** Allo-HSCT, Genetic polymorphism, MBL, Infection, Outcome

## Abstract

**Background:**

Mannose-binding lectin (MBL) is a key component of innate immunity. Low serum MBL levels, related to promoter polymorphism and structural variants, have been associated with an increased risk of infection. The aim of this work was to analyse the incidence and severity of infections and mortality in relation to the *MBL2* genotype and MBL levels in patients underwent allogeneic haematopoietic stem cell transplantation (Allo-HSCT).

**Results:**

This was a prospective cohort study of 72 consecutive patients underwent Allo-HSCT between January 2007 and June 2009 in a tertiary referral centre. Three periods were considered in the patients’ follow-up: the early period (0–30 days after Allo-HSCT), the intermediate period (30–100 days after Allo-HSCT) and the late period (> 100 days after Allo-HSCT). A commercial line probe assay for *MBL2* genotyping and an ELISA Kit were used to measure MBL levels. A total of 220 episodes of infection were collected in the 72 patients. No association between donor or recipient *MBL2* genotype and infection was found. The first episode of infection presented earlier in patients with pre-transplant MBL levels of < 1000 ng/ml (median 6d vs 8d, *p* = 0.036). MBL levels < 1000 ng/ml in the pre-transplant period (risk ratio (RR) 2.48, 95% CI 1.00–6.13), neutropenic period (0–30 days, RR 3.28, 95% CI 1.53–7.06) and intermediate period (30–100 days, RR 2.37, 95% CI 1.15–4.90) were associated with increased risk of virus infection. No association with bacterial or fungal disease was found. Mortality was associated with pre-transplant MBL levels < 1000 ng/ml (hazard ratio 5.55, 95% CI 1.17–26.30, *p* = 0.03) but not with *MBL2* genotype.

**Conclusions:**

Patients who underwent Allo-HSCT with low pre-transplant MBL levels presented the first episode of infection earlier and had an increased risk of viral infections and mortality in the first 6 months post-transplant. Thus, pre-transplant MBL levels would be important in predicting susceptibility to viral infections and mortality and might be considered a biomarker to be included in the pre-transplantation risk assessment.

## Background

Despite the improvement in diagnosis and therapy, invasive bacterial, virus and fungal infections are still some of the most important barriers to the success of allogeneic haematopoietic stem cell transplantation (Allo-HSCT) [1–3]. Mannose-binding lectin (MBL), a key component of innate immunity response, is a C-type serum lectin that binds microbial surface carbohydrates and mediates opsono-phagocytosis both directly and by activation of the lectin complement pathway [4, 5]. Mutations in the structural and regulatory sequence of the *MBL2* gene lead to inter-individual variations in serum MBL levels which have been associated with increased risk of infections [5–7]. Low serum MBL levels, related to promoter polymorphism and structural variants, have been associated with an increased risk of infection [7].

In patients with haematological malignancies receiving chemotherapy, increased susceptibility to infection and prolonged duration of febrile neutropenic episodes have been associated with MBL deficiency [8–10]. However, these observations were not consistent in all study cohorts [11–13]. In autologous HSCT [14–17] showed conflicting results regarding the influence of *MBL2* genotype and functional MBL deficiency on infection risk. In myeloablative, total body irradiation (TBI) -conditioned transplantation-, *MBL2* variants have been associated with an increased risk of infection [18]. Nonetheless, other studies involving Allo-HSCT showed contradictory results regarding the influence of *MBL2* polymorphisms and MBL deficiency on infection risk [18–25]. In addition, it is difficult to compare these different studies, because of some studies focused on MBL levels while others determined *MBL2* genotype, and even more because of different MBL cut-off levels and different *MBL2* genotype or haplotype combinations that are considered deficient [14–25]. Furthermore, over the years, research on MBL in Allo-HSCT has addressed the question of whether or not MBL is associated with their prognosis without reaching clear conclusions [13].

The aim of our study was to investigate the incidence, severity of infections, and mortality according to *MBL2* genotype and MBL levels in a prospective cohort of myeloablative and non-myeloablative Allo-HSCT patients carrying out a follow up from the pre-transplant conditioning to 6 months after the transplantation.

## Results

### Infections

From February 28th, 2007 to June 30th, 2009, 77 consecutive Allo-HSCT patients were included in the study. Five patients were excluded for not following the sampling protocol. The main demographic data are shown in Table 1 and supplementary Table 1.

**Table 1:** Characteristics of 72 patients with Allo-HSCT

Patients	
Age (median years, range)	44 (13–63)
Sex (male)	35 (48.6%)
Haematologic disease	
Acute Myeloblastic Leukaemia	18 (25.0%)
Acute Lymphoblastic Leukaemia	13 (18.1%)
Myelodysplastic syndrome	8 (11.1%)
Myeloproliferative syndrome	8 (11.1%)
Hodgkin Lymphoma	6 (8.3%)
Multiple Myeloma	5 (6.9%)
Chronic Lymphoproliferative Disorder	7 (9.7%)
NHL^a^	3 (4.2%)
Bone Marrow Aplasia	3 (4.2%)
Waldeström Disease	1 (1.4%)
Type of Transplant	
Myeloablative	38 (52.8%)
Non-myeloablative	34 (47.2%)
Conditioning	
Busulfan + Cyclophosphamide + /−ATG	23 (32.0%)
Fludarabine + Melphalan + /−ATG	18 (25.0%)
Radiotherapy + Cyclophosphamide + /−ATG	12 (16.7%)
Busulfan + fludarabine	11 (15.3%)
Others	8 (11.1%)
MBL levels (ng/mL) (median, IQR^b^)	
Pre-transplant	1135 (2918–466)
Early period	1997.5 (4056–464)
Intermediate period	1665 (3765–337.3)
Late period	1264 (3219.5–468.4)

^a^NHL: Non-Hodgkin’s Lymphoma

^b^IQR: interquartile range (75th–25th percentiles)

A total of 220 episodes of infection were collected, and 150 of them microbiologically documented (68.2%). There were 4 (6.6%) patients who had no infections during the follow up. Seventy-eight infections occurred in the early period, 52 in the intermediate period and 20 in the late period (Table 2). The average number of episodes per patient was 3 (SD = 2.1), with a minimum of 1 and a maximum of 9. The number of infections according to the conditioning regimen was 82.4% in myeloablative and 76.3% in non-myeloablative (*p* = 0.36). However, infections started later in patients with non-myeloablative allo-HSCT (median of 6 days in myeloablative conditioning regimen versus 9 days non-myeloablative, *p* = 0.018), with a significant HR = 1.76 (95% CI 1.06–2.71).

**Table 2:** Microbiologically documented episodes of infection

Microorganisms	Periods			
	Neutropenic^a^	Intermediate^b^	Late^c^	Total ^d^
	N (%)	N (%)	N (%)	N (%)
Bacteria	63 (80.8)	27 (51.9)	11 (55.0)	101 (67.3)
Gram-positive	39 (50.0)	8 (15.4)	3 (15.0)	50 (33.3)
Gram-negative	20 (25.6)	14 (26.9)	7 (35.0)	41 (27.3)
Polymicrobial	4 (5.1)	5 (9.6)	1 (5.0)	10 (6.7)
Virus	13 (16.7)	22 (42.3)	4 (20.0)	39 (26.0)
CMV^e^	5 (6.4)	18 (34.6)	4 (20.0)	27 (18.0)
Others non CMV^f^	8 (10.3)	4 (7.7)		12 (8.0)
Fungi	2 (2.6)	2 (3.8)	4 (20.0)	8 (5.3)
Candida	2 (2.6)		1 (5.0)	3 (2.0)
*Aspergillus*		2 (3.8)	3 (15.0)	5 (3.3)
*P. jirovecii*			1 (5.0)	1 (0.7)
Protozoa		1 (1.9)		1 (0.7)
Total	78	52	20	150

^a^0–30 days after Allo-HSCT; ^b^30–100 days after Allo-HSCT; ^c^ > 100 days after Allo-HSCT

^d^Total episodes of infection; ^e^CMV: Cytomegalovirus; ^f^Others non CMV virus: neutropenic period (3 herpesvirus, 2 polyomavirus, 1 herpes simplex virus type 1, 1 respiratory syncytial virus and 1 rotavirus), intermediate period (2 herpes simplex virus type 1, 1 adenovirus and 1 polyomavirus)

### Donors and recipients *MBL2* genotypes and infection

*MBL2* genotypes were analysed in 61 (84.7%) recipients and 38 (52.8%) donors. Recipients and donors were divided into two groups, each according to their *MBL2* genotype: 1) homozygous donors (AA) for wild type *MBL2* genotype (*n* = 24) and AO/OO donors (*n* = 14), which included heterozygous donors (AO) (*n* = 13) and homozygous mutant donors (OO) (n = 1); and 2) recipients homozygous (AA) for wild type *MBL2* genotype (*n* = 34) and AO/OO recipients (*n* = 27), which included heterozygous recipients (AO) (*n* = 23), and homozygous mutant recipients (OO) (*n* = 4) (supplementary Table 2). We did not find a significant increase of microbiologically documented Gram-positive (RR 1.71; CI 95% 0.90–3.28), Gram-negative (RR 1.56 CI 95% 0.90–2.69), virus (RR 1.50 CI 95% 0.69–3.24) or fungal infections (RR 1.00 CI 95% 0.03–2.66) in patients whose donors had low-producer *MBL2* genotypes (AO and OO genotypes). These findings did not differ when we tested different combinations of patient /donor *MBL2* genotypes.

Recipients with low-producer *MBL2* genotypes (AO and OO genotypes) had a tendency to have more episodes of infection (2.42, SD = 1.84) than those with *MBL2* wild-type genotype (AA genotypes) (1.74, SD = 1.04), *p* = 0.05. However, no association between *MLB2* polymorphisms and the risk of Gram-positive (RR 1.02, 95% CI 0.60–1.74), Gram-negative infections (RR 0.89, 95% CI 0.52–1.52), viruses (RR 0.63, 95% CI 0.30–1.34) or fungal infections (RR 0.31, 95% CI 0.03–2.66) was found during the first 6 months after the transplant. Supplementary Table 3 shows patients with *MBL2* genotypes and risk of infections.

### Serum MBL levels and infection

**Fig. 1:**
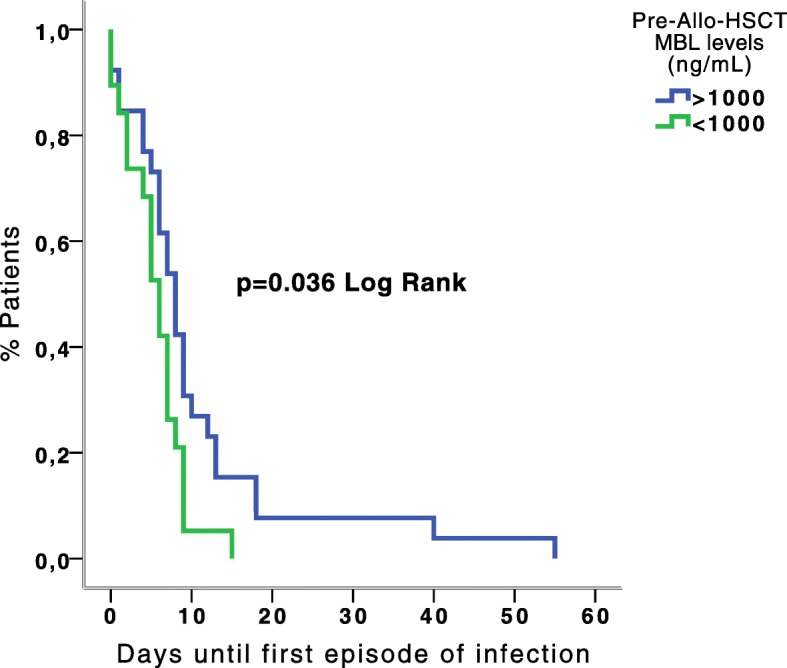
Timing of the first post-transplant episode of infection in patients according to pre-Allo-HSCT MBL levels

**Fig. 2:**
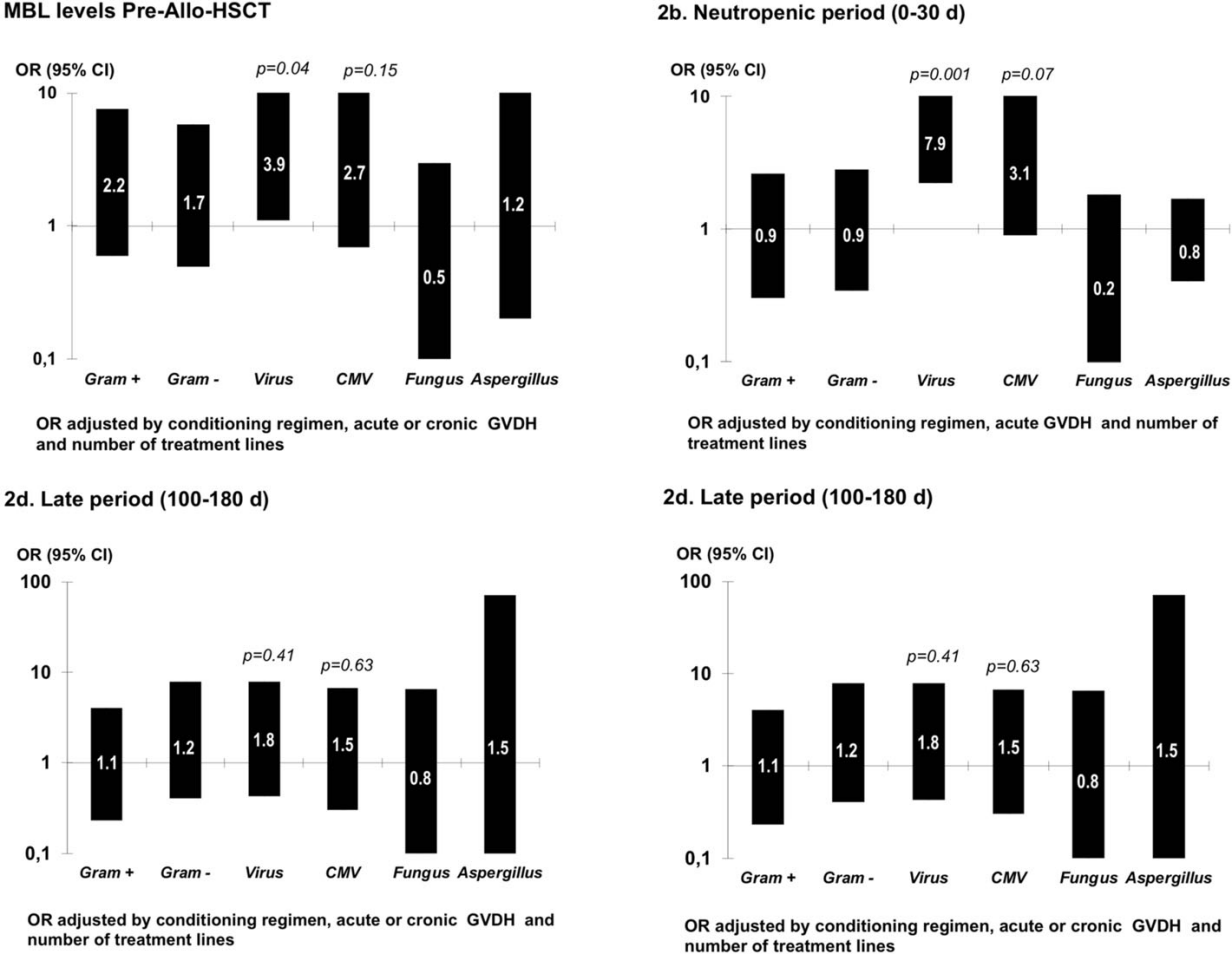
MBL levels < 1000 ng/mL and adjusted risk of bacterial, virus and fungal infections in 72 patients with Allo-HSCT. Figure 2a, MBL levels pre-transplant (before conditioning); Fig. 2b, neutropenic period (0–30 d); Fig. 2c, intermediate period (30-100d) and Fig. 2d, late period (100–180 d)

Patients with MBL levels < 1000 ng/ml in the pre-transplant period presented the first post-transplant infection episode earlier (median 6d vs. 8d, *p* = 0.036, HR adjusted by conditioning regimen 1.72, 95% CI 0.91–3.25) (Fig. 1). Additionally, a significant association was found between MBL levels < 1000 ng/mL in the pre-transplant (before conditioning) and the risk of virus infection after Allo-HSCT, in the early (RR 2.48, 95% CI 1.00–6.13), neutropenic (RR 3.28, 95% CI 1.53–7.06) and intermediate periods (RR 2.37, 95% CI 1.15–4.90). However, this finding was not observed in the late period. Among the virus infections, cytomegalovirus (CMV) was the most frequent infection (Table 2), and patients with low serum MBL levels had more episodes of CMV disease than those with normal levels, although these differences were not statistically significant. In the same way, no significant differences were observed between MBL levels and bacterial or fungal infections. The adjusted ORs are shown in Fig. 2 (2a, 2b, 2c, 2d).

### Effect of MBL levels and infections on survival

**Fig. 3:**
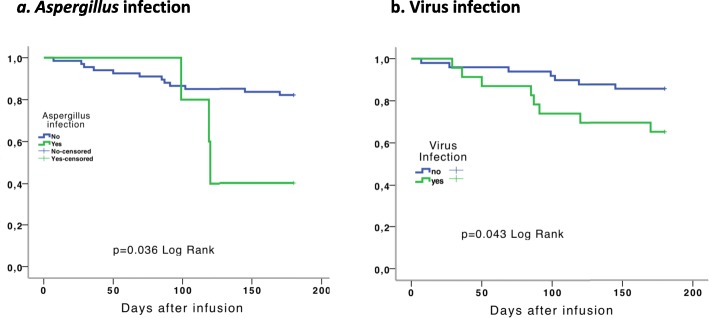
Survival time according to presence of *Aspergillus* and viral infections over the 6 months follow-up. Figure 3**a**, *Aspergillus* infection; 3**b**, virus infections

**Fig. 4:**
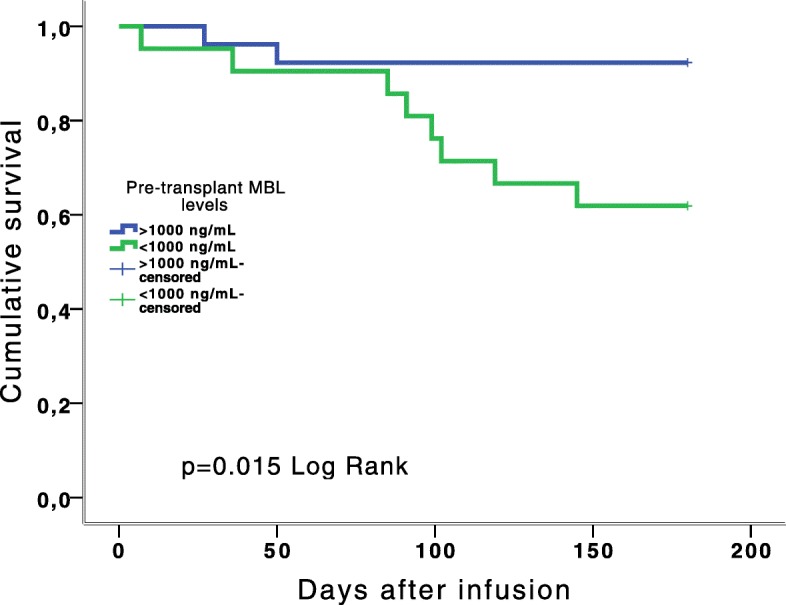
Survival time by pre-Allo-HSCT MBL status over the 6 months follow-up

Sixteen (22.2%) patients died (7 with myeloablative conditioning and 9 non-myeloablative). Causes of death in 12 patients with MBL levels < 1000 ng/mL , were: 6 patients died due to an infection (2 pulmonary aspergillosis,  2 *Pseudomonas aeruginosa bacteremia*, 1 *Klebsiella pneumoniae bacteremia* and 1 *Pneumocystis jiroveci* pneumonia), 2 patients because of graft versus host disease (GVHD), 2 patients due to a progression of the disease and 1 patient died due to a hemorrhage. Of the 4 patients who died and had MBL levels> 1000  ng/mL, 3 died due to an infection (1 pulmonary aspergillosis, 1 *Pseudomonas aeruginosa* bacteremia and another one *E. coli* and *Nocardia* spp. bacteremia) and 1 due to a multiorgan failure of unknown cause. *Aspergillus* and viral infections were associated with mortality (Figs. 3 and 4). It was estimated that patients who developed *Aspergillus* infection had a 3.56-fold higher death hazard than patients who did not (95% CI 0.99–12.70, *p* = 0.05). Mean survival time over the 6-month follow-up period in patients with and without *Aspergillus* infection was 139.60 days (95% CI 109.95–169.25) and 161.16 days (95% CI 150.40–171.92 respectively, *p* = 0.036) (Fig. 3a). Similarly, patients with a virus infection had a 2.73-fold higher death hazard than patients who did not (95% CI 0.99–7.55, *p* = 0.052). Mean survival time over the 6-month follow-up period in patients with and without virus infection was 146.43 days (95% CI 124.90–167.96) and 165.87 days (95% CI 155.03–176.72 respectively, *p* = 0.043) (Fig. 3b).

*MBL2* genotypes were available in 12 out of the 16 patients who died (3 YA/YA, 3 XA/YA, 4 YA/O, 1 XA/O and 1 O/O). There was no relationship between the recipient’s low producer *MBL2* genotypes and mortality (RR 1.26, 95% CI 0.46–3.47, *p* = 0.44). However, recipients with pre-transplant MBL levels < 1000 ng/mL had a significantly higher mortality rate than those with levels > 1000 ng/mL (HR 5.55, 95% CI 1.17–26.30, *p* = 0.03). In addition, the mean survival time of patients with pre-transplant MBL levels < 1000 ng/mL was significantly lower (143.95 days; 95% CI 121.49–166.41) than those with levels > 1000 ng/mL (169.15 days; 95% CI 154.57–183.66, *p* = 0.015). A Kaplan–Meier plot of survival by MBL status is shown in Fig. 4.

## Discussion

This prospective study focused on analysing the influence of *MBL2* polymorphisms and MBL serum levels in the incidence of infections and outcome of myeloablative and non-myeloablative Allo-HSCT patients. Patients were followed from the conditioning until 180 days after transplant. To date, the studies describing the incidence of infections in Allo-HSCT related to *MBL2* gene variants and MBL levels have shown conflicting results.

The results of our study indicate that donor and/or recipient *MBL2* genotype was not associated with an increased number of infections by Gram-positive, Gram-negative, virus or fungal infections in the six months following transplantation. These findings contrast with those of Mullighan et al. [22] and Granell et al. [20], which showed that the *MBL2* genotype of both recipients and donors influenced the risk of bacterial and invasive fungal infections, respectively, after Allo-HSCT. However, our results did support those found by Neth et al. [23], which suggested that donors’ *MBL2* genotype did not influence infection rates, and the results of Rocha et al. [21], who examined 107 HLA identical sibling Allo-HSCT recipients with myeloablative conditioning and found no association between *MBL2* gene polymorphisms and the development of bacterial, viral or fungal infections during the first 180 days after transplant. Currently, there are conflicting data about whether MBL deficiency is a good predictor of increased risk of infection. In our study, patients with pre-transplant MBL levels < 1000 ng/mL presented the first episode of infection earlier on. Similar results have been published about neutropenic post-chemotherapy patients, in whom low MBL levels have also been described to be associated with earlier infections [9, 25]. In addition, patients with MBL levels < 1000 ng/mL in the pre-transplant and during the first 100 days after the transplant, had a significantly increased risk (2 to 8 times higher) of viral infection, mainly for CMV infection, although in this case the difference did not reach statistical significance. Our results are consistent with previously reported data regarding the association of viral infection and MBL deficiency [26–28]. MBL binds to influenza A virus, and therefore, it would inhibit haemagglutinin activity and infectivity [29]. Furthermore, MBL deficiency has also been related with herpes simplex virus (HSV)-2 infection and with CMV infection and reactivation in kidney and lung transplant recipients [28–30]. Nevertheless, different results have reported the association of MBL deficiency with viral infections in Allo-HSCT recipients [18, 19, 23]. Chaudhry et al. [19] did not find an association between MBL levels < 400 ng/mL and viral infections in 110 paediatric Allo-HSCT recipients. However, Neth et al. [23] found an association between MBL levels < 400 ng/mL and HSV/varicella zoster virus (VZV) reactivation in 131 Allo-HSCT recipients, and Osthoff et al. [18] showed an independent association between MBL levels < 1000 ng/mL and the occurrence of severe herpes virus infections in 44 Allo-HSCT recipients. These findings would suggest an important role of the innate immunity in the control of an important viral complication after Allo-HSCT transplantation.

Likewise, there are conflicting results or contradictory data about whether *MBL2* genotypes or MBL deficiency could be used as mortality predictors in HSCT patients [13, 16, 17, 30, 31]. In the present study, the cause of death in all patients was infection, predominantly viral and *Aspergillus* infection. Recent report shows association of fungal invasive disease with death in patients with haematologic malignancy [13]. We did not find a relation between the recipient or donor *MBL*2 genotype and mortality. Our findings are in agreement with those of Mulligan et al. [17], who did not find a relationship between mortality and *MBL2* genotype, and disagree with our previous results in autologous HSCT [16] or those reported by other groups with immunosuppressed patients who had low-producing *MBL2* genotypes [30, 31]. On the other hand, we found that MBL levels < 1000 ng/mL could be a biomarker predictor of death since mortality was associated with low pre-transplant MBL levels. Similar findings have been published in patients with solid organ transplant, pneumonia or pneumococcal sepsis [32–34]. Low recipient serum MBL levels were associated with increased inflammation and apoptosis, [32] and have been described as an independent risk factor for poor prognosis in patients with pneumonia [35] and were associated with an increased severity of pneumococcal sepsis [34]. A meta-analysis published by Goa et al. in patients with sepsis showed a correlation between decreased MBL levels and development of sepsis, which the authors attributed to a possible interaction between coagulation patterns, proinflammatory cytokines and the complement system [35]. To date, there is a shortage of reports regarding the effects of MBL levels on the clinical outcome of Allo-HSCT patients. A recent retrospective study by Riwes et al. [13] reported no association between low MBL levels and an increased risk of invasive fungal disease or overall survival in patients with haematologic malignancy.

This study presents some limitations. First that it is a single-centre study, with potential for uncontrolled selection biases and with small simple size. Nevertheless, is a prospective study without variation in the methodologies, something that does not occur when drawing patients from multiple centres. To avoid information bias, this was collected prospectively; the sample analysis was blinded for the presence or absence of infection and the measurement of the effect (presence of infection); the infectious disease physicians using the same diagnostic criteria (according to the IDSA criteria) carried out the analysis; and physicians responsible for the monitoring and diagnosis of infections were masked with respect to the presence of genetic polymorphisms and/or MBL levels. Second, this study finished time ago and the viral o fungal prophylaxis of infection could have been changed and could influence our results. However, the procedure of HSCT is totally similar to the performed in a Hematology Service at present and to our knowledge, there are no extensive studies in the last years regarding the role of MBL in Allo-HSCT; in addition, our study has an important value because of a prospective cohort that included patients with myeloablative and non-myeloablative conditioning (the majority of studies on MBL and polymorphisms in patients with Allo-HSCT studied only those patients with myeloablative conditioning), this allows us to study a more heterogeneous population that is closer to the reality of the patients admitted to the Hematology Service. On the other hand, previous retrospective published studies on the role of innate immunity to infection or mortality in HSCT patients included old cohorts [14, 23], when probably viral and aspergillus prophylaxis were different to ours, with similar findings.

## Conclusions

In summary, our results would suggest that low pre-transplant MBL levels, but not *MBL2* genotype, could predispose patients undergoing Allo-HSCT to have the first episode of infection earlier and to increase the risk of viral infection. This would support the hypothesis that phenotype might be more important than genotype in predicting susceptibility to viral infections since this association would be related with MBL levels. Moreover, low pre-transplant MBL levels and no *MBL2* genotype would predict mortality in Allo-HSCT patients. Considering our findings, we could think that determination of the pre-Allo-HSCT MBL serostatus should be considered a useful biomarker for viral infection and mortality and should be included in the pre-transplantation risk assessment. However, given the contradictory findings that exist regarding the MBL studies after Allo-HSCT a larger study is required to replicate or refute our findings.

## Methods

A prospective cohort study of all patients who received an Allo-HSCT was carried out between February 2007 and June 2009 at a tertiary care university hospital (1000-bed). All Allo-HSCT patients were > 13 years old, and none of them presented any primary or secondary immunodeficiencies (solid neoplasms, HIV infection, autoimmune diseases). Patients were followed for a period of six months, after inclusion in the study.

### Endpoint definition

Intercurrent infections were prospectively collected from the first day after infusion (day+ 1) and for the next 180 days after transplantation. We considered 3 periods: the early period (0–30 days after Allo-HSCT), the intermediate period (30–100 days after Allo-HSCT) and the late period (> 100 days after Allo-HSCT). Infections were recorded by the clinical research associates from the institution using a standardized data collection form, following the protocols of the Hematology Department for autologous stem cell transplantation (ASCT) and Allo-SCT [17], in agreement with the clinical guidelines of the Infectious Diseases Society of America (IDSA) [36]. Prophylactic measures included isolation in high-pressure rooms with air filters, the administration of intravenous acyclovir at prophylactic doses from day 3, anti-bacterial prophylaxis with cotrimoxazole from day 30 after transplant and antifungal prophylaxis with fluconazole or caspofungin.

As previously described [17], we followed the protocols of the Hematology Department for definitions of an infectious episode (microbiologically or a clinically documented infection), major infection (either confirmed sepsis or systemic inflammatory response syndrome with highly suggestive radiographic or clinical features who received specific antibiotherapy); severe herpes virus infection (as an invasive viral infection requiring treatment and hospitalization); clinically significant CMV infection/reactivation (positive CMV blood culture or CMV-pp65 antigenemia assay or polymerase chain reaction (PCR) for CMV DNA (> 400 copies/mL, COBAS Amplicor CMV Monitor, Roche Diagnostics) and symptoms of organ dysfunction were required to fulfil the criteria of a clinically significant CMV infection/reactivation [37]). Episodes of asymptomatic CMV reactivation, local HSV reactivation, primary VZV, dermatological varicella zoster reactivation, patients with a single positive blood culture with contaminant skin bacteria, upper respiratory tract infections and culture-negative interstitial pneumonitis were excluded.

### *MBL2* genotyping

Blood samples from transplant patients was collected the day before the start of conditioning. In the case of donors, it was collected on the day of infusion in related donors. Eighteen unrelated donors were excluded for this analysis. There were 16 related donors in whom the sample could not be obtained; therefore, the determination of the genotype in the donors was finally performed in 34 patients. As previously described [17], genomic DNA from patients and controls was purified from blood samples by using the Maxwell 16 Genomic DNA Purification system (Promega Biotech Ibérica S.L., Madrid, Spain) and for *MBL2* gene amplification and genotyping, the INNO-LiPA *MBL2* (Innogenetics Diagnostica Iberia S.L.U, Barcelona, Spain) was used, following the manufacturer’s instructions. We analysed the 6 variations in the human *MBL2* gene (−550G > C, −221G > C, +4C > T, R52C, G54D, and G57E), the seven common haplotypes and the 28 possible resulting diplotypes. Exonic structural variants (A: normal allele, and B, C and D: mutant alleles, which together were designated allele O) and promoter polymorphisms (H/L, Y/X and P/Q) were analysed. Variants YA/YA, XA/XA and YA/XA were classified as A/A. Those with any mutation in the exon such as YA/O and XA/O were classified as A/O, and homozygous individuals with structural variants in the exon were considered O/O. Thus, patients were divided into two groups according to their *MBL2* genotype: 1) normal homozygous patients (AA), and 2) patients with AO and OO genotypes, who as described earlier, were considered to be lower MBL producers [15].

### MBL serum levels measurement

Serum samples were used for the determination of MBL levels. These samples were obtained from patients before conditioning, weekly during the first month after the transplant and monthly during the following 5 months after the transplant. If an episode of infection occurred, biweekly serum was collected until the resolution of the episode.

Serum samples were collected and stored at − 80 °C until analysis. Quantification of functional MBL was performed by an investigator blinded to any recipient data using a commercially available ELISA Kit (MBL Oligomer ELISA Kit, Bio Porto Diagnostics A/S, Copenhagen, Denmark) following manufacturer recommendations. Functional MBL deficiency was defined as serum levels below 1000 ng/mL [18].

### Statistical analysis

As previously described [17], statistical analysis was performed, as required, using χ2 and Fisher’s exact test when necessary, as well as Student’s t test, or the Mann-Whitney test. A two-tailed *p* < 0.05 was considered statistically significant. To analyse the association between *MBL2* genotype and MBL levels, relative risks (RR) and adjusted odds ratios (OR) and their 95% confidence intervals (CI) were calculated; the risk of concomitant infections was the dependent variable (overall and by microorganisms). The effect of MBL levels pre-transplant (before the start of conditioning) and after transplantation (early, intermediate and late periods) on the risk of infection, was analysed. Multivariate analysis was performed using logistic regression: a regression model was constructed for each type of infection, with infection-related variable being the outcome and the independent variable being the *MBL2* genotypes and MBL levels, adjusted for sex, age, conditioning regimen, acute or chronic GVHD, and number of treatment lines. We used Kaplan-Meyer survival analysis with the log-rank test for comparisons between survival time and time to event or time until a certain event (1st infection). To estimate survival rate over time a Cox regression was applied as a function of several covariates. Hazard ratios (HR) and their 95% CI were calculated. Data were analysed using Stata (SE 10.0, Stata Corporation, College Station, TX) and SPSS (v19) (SPSS Inc., Chicago, IL) statistical software.

## Supplementary information

**Additional file 1: Table S1.** Sociodemographic and medical conditions of each patient with Allo-HSCT. **Table S2.** Genotypes of MBL according to mutations of the exon and promoter, exon and X / Y of the promoter and according to exon mutations in patients and donors. **Table S3.** Patients´ *MBL2* genotypes and risk of infections.

## Data Availability

All data used and analyzed during the present study will be available from the corresponding author on reasonable request.

## Abbreviations

Allo-HSCT: Hematopoietic allogeneic stem cell transplantation
CI: Confidence Interval
CMV: Cytomegalovirus
DNA: Deoxyribonucleic Acid
GVHD: Graft Versus Host Diseases
HCV: Hepatitis C Virus
HIV: Human Immunodeficiency Virus
HLA: Human leukocyte antigen
HR: Hazard Ratio
HSV: Herpes simplex virus
IDSA: Infectious Diseases Society of America
MBL: Mannose-Binding Lectin
PCR: Polymerase Chain Reaction
RR: Risk Ratio
SCT: Stem cell transplantation
VZV: Varicella zoster virus
