# Supplementary material for: Low pre-transplant levels of mannose-binding lectin are associated with viral infections and mortality after haematopoietic allogeneic stem cell transplantation
Source: BMC Immunol. 2019 Nov 9;20:40. doi: 10.1186/s12865-019-0318-8 (PMC6842494; doi:10.1186/s12865-019-0318-8)
Supplement: Supplementary file 1 — Additional file 1: Table S1. Sociodemographic and medical conditions of each patient with Allo-HSCT. Table S2. Genotypes of MBL according to mutations of the exon and promoter, exon and X / Y of the promoter and according to exon mutations in patients and donors. Table S3. Patients´ MBL2 genotypes and risk of infections. [file 12865_2019_318_MOESM1_ESM.docx]

**Table S1.** Sociodemographic and medical conditions of each patient with Allo-HSCT.

| **Patients** | **Age range (yr.)** | **Hematologic disease** | **Conditioning** | **Type of transplant** |
| --- | --- | --- | --- | --- |
| 1 | 31-40 | Myeloproliferative syndrome | Busulfan + Cyclophosphamide | Myeloablative |
| 2 | 21-30 | Acute Lymphoblastic Leukemia | Radiotherapy + Cyclophosphamide | Myeloablative |
| 3 | 21-30 | Acute Lymphoblastic Leukemia | Radiotherapy + Cyclophosphamide | Myeloablative |
| 4 | 21-30 | Acute Myeloblastic Leukemia | Fludarabine + Melphalan +ATG | Non-myeloablative |
| 5 | 31-40 | Hodgkin Lymphoma | Fludarabine + Melphalan | Non-myeloablative |
| 6 | 31-40 | Hodgkin Lymphoma | Others | Myeloablative |
| 7 | 21-20 | Acute Myeloblastic Leukemia | Radiotherapy + Cyclophosphamide | Myeloablative |
| 8 | 41-50 | Bone Marrow Aplasia | Others | Myeloablative |
| 9 | 41-50 | Acute Myeloblastic Leukemia | Fludarabine +Busulfan | Myeloablative |
| 10 | 41-50 | Multiple Myeloma | Fludarabine + Melphalan | Non-myeloablative |
| 11 | 31-40 | Hodgkin Lymphoma | Fludarabine + Melphalan | Non-myeloablative |
| 12 | 51-60 | Acute Lymphoblastic Leukemia | Busulfan + Cyclophosphamide | Myeloablative |
| 13 | 41-50 | Myeloproliferative syndrome | Busulfan + Cyclophosphamide | Myeloablative |
| 14 | 41-50 | Myeloproliferative syndrome | Fludarabine +Busulfan | Non-myeloablative |
| 15 | 51-60 | Chronic Lymphoproliferative Disorder | Fludarabine + Melphalan | Non-myeloablative |
| 16 | < 20 | Bone Marrow Aplasia | Others | Non-myeloablative |
| 17 | 41-50 | Myeloproliferative syndrome | Busulfan + Cyclophosphamide +ATG | Myeloablative |
| 18 | 41-50 | Myeloproliferative syndrome | Others | Non-myeloablative |
| 19 | 41-50 | Chronic Lymphoproliferative Disorder | Fludarabine + Melphalan | Non-myeloablative |
| 20 | 41-50 | Myeloproliferative syndrome | Fludarabine +Busulfan | Non-myeloablative |
| 21 | 51-60 | Myeloproliferative syndrome | Fludarabine +Busulfan | Non-myeloablative |
| 22 | 51-60 | Acute Myeloblastic Leukemia | Busulfan + Cyclophosphamide + ATG | Myeloablative |
| 23 | 51-60 | Acute Myeloblastic Leukemia | Busulfan + Cyclophosphamide | Myeloablative |
| 24 | 51-60 | Acute Lymphoblastic Leukemia | Radiotherapy + Cyclophosphamide | Myeloablative |
| 25 | 41-50 | Myelodysplastic syndrome | Busulfan + Cyclophosphamide | Myeloablative |
| 26 | 61-70 | Multiple Myeloma | Fludarabine + Melphalan | Non-myeloablative |
| 27 | 31-40 | Myeloproliferative syndrome | Busulfan + Cyclophosphamide | Myeloablative |
| 28 | 51-60 | Acute Myeloblastic Leukemia | Fludarabine +Busulfan | Non-myeloablative |
| 29 | 31-40 | Acute Lymphoblastic Leukemia | Radiotherapy + Cyclophosphamide + ATG | Myeloablative |
| 30 | 41-50 | Acute Myeloblastic Leukemia | Busulfan + Cyclophosphamide | Myeloablative |
| 31 | 61-70 | Chronic Lymphoproliferative Disorder | Others | Non-myeloablative |
| 32 | 21-30 | Acute Myeloblastic Leukemia | Fludarabine +Busulfan | Myeloablative |
| 33 | 41-50 | Acute Lymphoblastic Leukemia | Radiotherapy + Cyclophosphamide | Myeloablative |
| 34 | 51-60 | Acute Myeloblastic Leukemia | Busulfan + Cyclophosphamide + ATG | Myeloablative |
| 35 | 61-70 | Myelodysplastic syndrome | Fludarabine +Busulfan | Non-myeloablative |
| 36 | 21-30 | Hodgkin Lymphoma | Fludarabine + Melphalan + ATG | Non-myeloablative |
| 37 | 61-70 | Chronic Lymphoproliferative Disorder | Others | Non-myeloablative |
| 38 | 21-30 | Acute Myeloblastic Leukemia | Busulfan + Cyclophosphamide | Myeloablative |
| 39 | 31-40 | Hodgkin Lymphoma | Fludarabine + Melphalan | Non-myeloablative |
| 40 | 31-40 | Myelodysplastic syndrome | Busulfan + Cyclophosphamide | Myeloablative |
| 41 | 61-70 | Acute Myeloblastic Leukemia | Busulfan + Cyclophosphamide | Myeloablative |
| 42 | 51-60 | Multiple Myeloma | Fludarabine + Melphalan | Non-myeloablative |
| 43 | 41-50 | Non-Hodgkin's Lymphoma | Fludarabine + Melphalan + ATG | Non-myeloablative |
| 44 | 21-30 | Myelodysplastic syndrome | Busulfan + Cyclophosphamide | Myeloablative |
| 45 | 31-40 | Acute Lymphoblastic Leukemia | Radiotherapy + Cyclophosphamide | Myeloablative |
| 46 | <20 | Acute Lymphoblastic Leukemia | Radiotherapy + Cyclophosphamide | Myeloablative |
| 47 | 21-30 | Non-Hodgkin's Lymphoma | Fludarabine + Melphalan +ATG | Non-myeloablative |
| 48 | 51-60 | Waldenstrom Disease | Radiotherapy +ATG | Non-myeloablative |
| 49 | 31-40 | Chronic Lymphoproliferative Disorder | Radiotherapy + ATG | Non-myeloablative |
| 50 | 21-30 | Acute Myeloblastic Leukemia | Busulfan + Cyclophosphamide | Myeloablative |
| 51 | 51-60 | Non-Hodgkin's Lymphoma | Fludarabine + Melphalan | Non-myeloablative |
| 52 | 31-40 | Acute Lymphoblastic Leukemia | Radiotherapy + Cyclophosphamide | Myeloablative |
| 53 | 31-40 | Acute Myeloblastic Leukemia | Busulfan + Cyclophosphamide | Myeloablative |
| 54 | 31-40 | Acute Lymphoblastic Leukemia | Radiotherapy + Cyclophosphamide | Myeloablative |
| 55 | 21-30 | Myelodysplastic syndrome | Busulfan + Cyclophosphamide | Myeloablative |
| 56 | 31-40 | Myelodysplastic syndrome | Busulfan + Cyclophosphamide | Myeloablative |
| 57 | 41-50 | Myelodysplastic syndrome | Busulfan + Cyclophosphamide | Myeloablative |
| 58 | 61-70 | Acute Myeloblastic Leukemia | Busulfan + Cyclophosphamide | Myeloablative |
| 59 | 51-60 | Myelodysplastic syndrome | Fludarabine +Busulfan | Non-myeloablative |
| 60 | 51-60 | Acute Lymphoblastic Leukemia | Busulfan + Cyclophosphamide | Myeloablative |
| 61 | 61-70 | Acute Myeloblastic Leukemia | Fludarabine +Busulfan | Non-myeloablative |
| 62 | 51-60 | Acute Myeloblastic Leukemia | Busulfan + Cyclophosphamide + ATG | Myeloablative |
| 63 | 41-50 | Acute Myeloblastic Leukemia | Fludarabine +Busulfan | Non-myeloablative |
| 64 | 51-60 | Multiple Myeloma | Fludarabine + Melphalan | Non-myeloablative |
| 65 | 51-60 | Chronic Lymphoproliferative Disorder | Fludarabine + Melphalan | Non-myeloablative |
| 66 | 31-40 | Acute Lymphoblastic Leukemia | Fludarabine +Busulfan | Non-myeloablative |
| 67 | 31-40 | Acute Myeloblastic Leukemia | Fludarabine +Busulfan | Non-myeloablative |
| 68 | 51-60 | Multiple Myeloma | Fludarabine + Melphalan | Non-myeloablative |
| 69 | 21-30 | Hodgkin Lymphoma | Fludarabine + Melphalan | Non-myeloablative |
| 70 | 51-60 | Chronic Lymphoproliferative Disorder | Fludarabine + Melphalan | Non-myeloablative |
| 71 | <20 | Bone Marrow Aplasia | Others | Myeloablative |
| 72 | <20 | Acute Lymphoblastic Leukemia | Radiotherapy + Cyclophosphamide +ATG | Myeloablative |

**Table S2.** Genotypes of MBL according to mutations of the exon and promoter, exon and X / Y of the promoter and according to exon mutations in patients and donors.

|  | **Pacients**  **(N=61) (%)** | **Donors**  **(N=38) (%)** |
| --- | --- | --- |
| **Promoter polymorphisms** | | |
| H | 46 (75.41) | 22 (57.89) |
| L | 49 (80.33) | 31 (81.58) |
| Y | 60 (98.36) | 36 (94.74) |
| X | 26 (42.62) | 22 (57.89) |
| P | 61 (100.00) | 38 (100.00) |
| Q | 19 (31.15) | 14 (36.84) |
| **Exon 1 structural variants** | | |
| A | 57 (93.44) | 37 (97.37) |
| B | 12 (19.67) | 5 (13.16) |
| C | 0 (0.00) | 0 (0.00) |
| D | 15 (24.59) | 8 (21.05) |
